# Identification and Characterization of a Newly Isolated Chitinase-Producing Strain *Bacillus licheniformis* SSCL-10 for Chitin Degradation

**DOI:** 10.1155/2020/8844811

**Published:** 2020-11-09

**Authors:** Abirami Sasi, Nagarajan Duraipandiyan, Kannan Marikani, Sugapriya Dhanasekaran, Noura Al-Dayan, Divya Venugopal

**Affiliations:** ^1^Department of Microbiology, Kamaraj College, Thoothukudi, India; ^2^Department of Zoology, Kamaraj College, Thoothukudi, India; ^3^Department of Zoology, V.H.N.S.N.College (Autonomous), Virudhunagar626001, TN, India; ^4^Department of Medical Lab Sciences, College of Applied Medical Sciences, Prince Sattam Bin Abdulaziz University, Wadi ad-Dawasir Campus, Saudi Arabia; ^5^Department of Medical Lab Sciences, College of Applied Medical Sciences, Prince Sattam Bin Abdulaziz University, Al Kharj, Saudi Arabia

## Abstract

Chitinases or chitinolytic enzymes have different applications in the field of medicine, agriculture, and industry. The present study is aimed at developing an effective hyperchitinase-producing mutant strain of novel *Bacillus licheniformis*. A simple and rapid methodology was used for screening potential chitinolytic microbiota by chemical mutagenesis with ethylmethane sulfonate and irradiation with UV. There were 16 mutant strains exhibiting chitinase activity. Out of the chitinase-producing strains, the strain with maximum chitinase activity was selected, the protein was partially purified by SDS-PAGE, and the strain was identified as *Bacillus licheniformis* (SSCL-10) with the highest specific activity of 3.4 U/mL. The induced mutation model has been successfully implemented in the mutant EMS-13 (20.2 U/mL) that produces 5-6-fold higher yield of chitinase, whereas the mutant UV-11 (13.3 U/mL) has 3-4-fold greater chitinase activity compared to the wild strain. The partially purified chitinase has a molecular weight of 66 kDa. The wild strain (SSCL-10) was identified as *Bacillus licheniformis* using 16S rRNA sequence analysis. This study explores the potential applications of hyperchitinase-producing bacteria in recycling and processing chitin wastes from crustaceans and shrimp, thereby adding value to the crustacean industry.

## 1. Introduction

Shrimp production in India was estimated to be 700,000 tons in 2019, with the state of Tamil Nadu being one of the main producers. The seafood industry makes a significant contribution to the global food supply providing an essential source of protein. The commercialization of this aquaculture has generated economic profits while the wastes produced by these industries have had an adverse effect on the ecosystem [1, 2]. The global fish production is estimated to rise from 154 million tons in 2011 to 186 million tons in 2030 [3]. Approximately 5% of shrimp wastes are processed into flours and extracts which form a base for animal feed [4]. Shrimp wastes consist of 40% chitin, a polysaccharide made up of N-acetylglucosamine units [5] and a significant primary resource for the source of bioactive molecules [6].

Chitin is degraded most frequently by the chemical pathway to generate oligosaccharides. However, this involves adverse consequences such as processing costs and harmful effects on the ecosystem with the use of highly corrosive chemical reagents [7, 8]. On the other hand, the biotechnological pathway is an ecofriendly approach [9] where chitinases (glycosyl-hydrolase proteins) play an important role in cleaving the *β*-1,4 bonds of the N-acetylglucosamine units resulting in chitin degradation [10].

Most of the common bacteria and fungi microbiota synthesize chitinolytic enzymes, and some unknown species effectively decompose this chitin polymer. Researchers have reported that marine environments are the principal source of chitinase-synthesizing microorganisms, mostly bacterial species [11] out of which only 4% of the strains are classified [12]. Among the genera that are identified as chitinase producers from the marine ecosystem include *Bacillus*, *Aeromonas*, *Serratia*, *Enterobacter*, *Erwinia*, *Chromobacterium*, *Flavobacterium*, *Arthrobacter*, and *Vibrio* [13]. Several other bacterial species that have been investigated for the production of chitinolytic enzymes are *Streptococcus*, *Clostridium*, and *Eubacterium* genera that were isolated from whale wastes [14]; *Bacillus licheniformis* from the liquid waste of the food industry [15]; *Streptomyces* and *Serratia* from the residues of crustaceans [16]; and *Bacillus amyloliquefaciens* and *Acinetobacter johnsonii* from shrimp residues [17]. Chitinase producers are rarely isolated from aquatic ecosystems as compared to terrestrial environments, as they are aerobic [12]. Screening for chitinase producers has become an important area for many researchers to help pave the way for degradation of shrimp residues in an economically feasible and ecofriendly manner. Therefore, the present investigation focused to isolate a chitinase-producing strain and to identify it using 16S rRNA sequencing. A mutant strain producing hyperchitinase was developed.

## 2. Materials and Methods

### 2.1. Organism, Media, and Culture Conditions

The strain *Bacillus licheniformis* (SSCL-10) that had been isolated from seafood industrial waste (shrimp shell dumping area) of Thoothukudi, India, and identified at the microbiology laboratory, V.H.N.S.N (autonomous), Virudhunagar, TN, India, has been used in this study. The culture conditions, optimal temperature, pH, and colloidal chitin concentration were followed according to an earlier report by Abirami et al. [7]. Chitin decomposing and antifungal properties have been identified and published [7, 18].

#### 2.1.1. Preparation of Colloidal Chitin and Colloidal Chitin Agar (CCA) Plates

Colloidal chitin was prepared according to [19]. Briefly, 5 g chitin powder (GRM1356-100G Hi-media, India) was added slowly to 60 L of (10 N HCl) concentrated HCl with continuous stirring at 4°C overnight. This mixture was added to 50% of 500 mL ice-cold ethanol at 25°C with continuous and vigorous stirring at 200 rpm overnight in the rotary shaker. After centrifugation at 10,000 rpm for 20 minutes, the precipitate was collected and washed with sterile distilled water until became neutral (pH 7.0). Collected colloidal chitin was freeze dried and stored at 4°C until use. The colloidal chitin agar plates were prepared according to our earlier report [7] by mixing 5 g of colloidal chitin with mineral salts (KH_2_PO_4_ 0.7 g, K_2_HPO_4_ 0.3 g, MgSO_4_·5H_2_O 0.5 g, FeSO_4_·7H2O 0.001 g, ZnSO_4_ 0.001 g, MnCl_2_ 0.001 g, and agar 20 g for 1 L with pH 8).

#### 2.1.2. Primary Screening of Chitin-Degrading Bacteria

Primary screening was performed by spot inoculating all the chitin-degrading bacterial isolates on CCA using toothpick heads of 2 mm diameter and incubated at room temperature. The zone of clearance due to chitin hydrolysis was recorded up to 5 days. The bacterial isolates producing clear zone over 5 mm alone were selected and subjected to secondary screening.

### 2.2. Determination of Specific Chitinolytic Activity

Chitinase activity was measured according to the method of Vyas and Deshpande [20]. Briefly, colloidal chitin was used as the substrate to measure chitinase activity. Enzyme solution (0.5 mL) was added to 1.0 mL of substrate solution (0.5% suspension of the colloidal chitin in a phosphate buffer (50 mM, pH 7.0)) incubated at 37°C for 15 minutes. After centrifugation, the supernatant was measured for reducing sugars by the dinitrosalicylic acid method and N-acetyl glucosamine was used as a standard [21]. One unit of chitinase activity was defined as the amount of enzyme required to liberate 1 *μ*mol of N-acetyl- D-glucosamine equivalent at 50°C h^−1^.

### 2.3. Determination of Shrimp Shell Degradation Using Chitinolytic Microorganisms

The biodegradation potential of *Bacillus licheniformis* (SSCL-10) was determined on shrimp shells. Briefly, the selected *Bacillus licheniformis* (SSCL-10) were grown in 0.5% colloidal chitin containing nutrient broth as inoculum. Fifty microliter of bacterial inoculum (0.5 OD) was inoculated to 1 g of shrimp shells in 5 mL minimal medium (pH 7) at 40°C in 100 rpm rotary shaker for 6 and 12 days. After incubation, based on the shrimp shell-degrading efficacy, the *Bacillus licheniformis* (SSCL-10) was selected for mutagenesis and maintained on nutrient agar slants for further studies.

### 2.4. Molecular Identification and Phylogenetic Tree Construction

#### 2.4.1. PCR Amplification

Molecular identification was done by 16S rRNA analysis. Briefly, high yield of chitinase producer *Bacillus licheniformis* (SSCL-10) reported in our earlier studies [7] was selected for the study. Bacterial genomic DNA of *Bacillus licheniformis* (SSCL-10) was extracted using the Insta Gene TM Matrix (Bio-Rad, cat-no. 7326030), according to the manufacturers' instruction. Then, a 16S rRNA subunit gene fragment was amplified by using 16S rRNA universal primers 27F (5′-AGAGTTTGATCMTGG CTCAG-3′) and the reverse primer 1492R (5′-TACGGYTACCTTGTTACGACTT-3′) [22] using MJ Research Peltier Thermal Cycler (Marshall Scientific, USA). PCR was performed in a 30 *μ*L reaction mixture (20 ng genomic DNA) under the following cycling conditions: 95°C for 2 min, followed by 35 cycles of 95°C for 1 min, 55°C for 1 min, and 72°C for 1 min, with a final incubation at 72°C for 10 min.

#### 2.4.2. 16S rRNA Gene Sequencing

The PCR product was detected using agarose gel electrophoresis and extracted using the Genei® Gel Extraction Kit (Bangalore Genei, India). Cycle sequencing was performed with the help of ABI3730xl DNA Analyzer with a BigDye Terminator Cycle Sequencing Kit v.3.1 (Applied Biosystems, Carlsbad, CA, USA). The primers 518F (5′-CCAGCAGCCGCGGTAATACG-3′) and 800R (5′-TACCAGGGTATCTAATCC-3′) were used for the sequencing reactions. The short sequence of the 16S rRNA gene was compiled using SeqMan (DNASTAR Inc.). The newly generated sequences were deposited in GenBank.

#### 2.4.3. Phylogenetic Analysis

To analyse the closest phylogenetic classification for the chitinase producer, the sequenced 16S rRNA genes were compared with BLAST (https://blast.ncbi.nlm.nih.gov/Blast.cgi) using the NCBI blast similarity search tool. The phylogenetic analysis of our sequence with the closely related sequence from the blast results was performed followed by multiple sequence alignment. The program MUSCLE 3.7 was used for multiple alignments of sequences [23]. The aligned sequences were cured using Gblocks 0.91b to eliminate poorly aligned positions and divergent regions (removes alignment noise) [24]. Finally, Phyml 3.0 aLRT program was used for phylogenetic analysis and HKY85 as the substitution model which is shown to be at least as accurate as other existing phylogeny programs using simulated data and the order of magnitude being faster. Tree Dyn 198.3 program was used for tree rendering [25].

### 2.5. UV Mutagenesis of *Bacillus licheniformis*

UV mutagenesis protocol was followed according to Vaidya et al. [26] with modifications. The wild-type *Bacillus licheniformis* (SSCL-10) was inoculated in nutrient broth and incubated at 37°C for 24 hours. One microlitre culture was exposed to short UV light wavelength (280 nm) from a distance of 60 cm (Philips TUV 30 W, G3018, Holland) with different time intervals (0, 2, 4, 6, 8, and 10 minutes) in an open glass petridish under dark conditions to protect from photoreactivation. Then, the culture was serially diluted on CCA plates and mutant strains were isolated from a low ratio of UV survivors. Mutants were screened and selected by observing higher zone of clearance (CZ) to colony size (CS) ratio and chitinase activity.

### 2.6. Chemical Mutagenesis of *Bacillus licheniformis*

Mutations were induced chemically by ethylmethane sulphonate (EMS) according to the method of Vaidya et al. [26] with modifications. The UV mutant strain was further mutated chemically by EMS to observe the effect on hyperchitinase production. The *Bacillus licheniformis* (SSCL-10) (UV-11) was inoculated in nutrient broth and incubated at 37°C for 24 hours. After incubation, the pellet was collected from 1 mL of culture medium and washed twice with sterile 0.1 M Tris-HCl buffer (pH 7.5) and suspended in equal volume of 0.1 M Tris-HCl buffer (pH 7.5). The pellet was collected and resuspended in half the volume of 0.1 M Tris-HCl buffer (pH 7.5). Cells were collected in 0.1 mL aliquots and kept on ice. A total of 50 *μ*g/mL of EMS was added to each tube and placed in a shaker water bath at 37°C for 30 minutes. The cells were washed and suspended in 1 mL with 0.1 M Tris-HCl buffer (pH 7.5), and the mutated sample was grown for 24 h. The induced mutants were serially diluted and plated on CCA plates. Mutants were screened and selected by observing for higher zone of clearance (CZ) to colony size (CS) ratio and chitinase activity.

### 2.7. Screening and Isolation of Hyperchitinase-Producing Mutant

After the mutagenesis process, the CCA plates were incubated at room temperature and examined up to 5 days for the zone of clearance (chitin hydrolysis). The colony size (CS) as well as the zone of clearance (CZ) was measured, and the CZ/CS ratios were measured and compared with the wild type. Higher CZ/CS colonies were subcultured on chitin agar slants and simultaneously inoculated into 100 mL nutrient broth containing 1% colloidal chitin and incubated at 30°C on a rotary shaker at 100 rpm for 48 h. The culture filtrate was collected and measured for chitinase activity.

### 2.8. Partial Purification of Chitinase

The crude enzyme was partially purified from the culture supernatant (wild, UV mutant, and EMS mutant strains) using 65% saturated ammonium sulfate (SAS) precipitation at 4°C in stirring according to the method of Akeed et al. [27]. Precipitation was done by centrifuging at 11,500 x g for 10 min at 4°C. The precipitate was resuspended in equal volume of sodium-acetate buffer (pH 6), and the protein concentration was estimated [28]. The isolate with the highest chitinase activity was purified on 12% polyacrylamide gel electrophoresis and stained using 0.5% Coomassie Brilliant Blue R250 (Sigma). A protein marker was also electrophoresed for size determination.

### 2.9. Statistical Analysis

Analysis of variance (ANOVA) was performed and the means were compared using Tukey tests (*p* < 0.05) on the specific chitinolytic activity using the SPSS 18.0 statistical software.

## 3. Results

### 3.1. Screening of the Wild-Type Strain

Our earlier study reported that the 16 bacterial strains isolated from shrimp residues expressed different hydrolysis halo sizes (zone of clearance over 5 mm in colloidal chitin agar medium) [8]. From these isolates, *Bacillus licheniformis* (SSCL-10) produced the highest chitinase activity that was used as the wild type (KCCD 201018) and compared with the mutant producers (Fig. S2). The SSCL-10 was catalogued in the institution collection of Kamaraj College Culture Depositary (KCCD 201018). Similarly, the mutant strains, UV-11 and EMS-13 were catalogued with the numbers KCCD 201807 and KCCD 201812, respectively.

**Figure 1:**
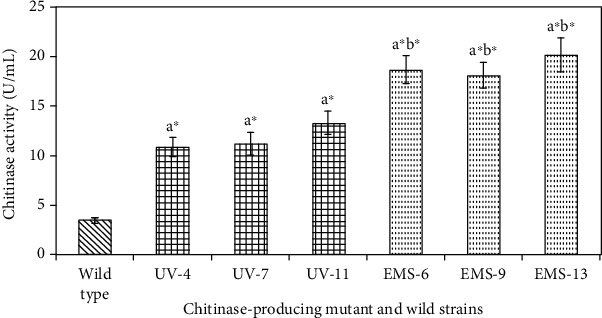
Specific chitinolytic activity of *Bacillus licheniformis* SSCL-10 wild type along with maximum chitinolytic activity of three mutants of UV and EMS strains. Values with a^∗^b^∗^ are significantly (*p* < 0.05) higher activity. a^∗^: wild type compared with UV and EMS mutant. b^6^: UV compared with EMS mutant.

The colony morphology of strain *Bacillus licheniformis* (SSCL-10) shows white, circular and convex shape with full borders and smooth texture. Microscopic examination showed rod-shaped, motile vegetative cells and endospore forming that was confirmed to belong to the *Bacillus* group (Fig. S3). The *Bacillus licheniformis* (SSCL-10) showed enzyme activity of 3.4 U/mL on the 4th day of incubation (Figure 1). Based on our previous study [8], the optimum concentration of colloidal chitin (1%), temperature 40°C, and pH 7 were used in this study for growth of both wild and mutant strains for the chitinase production.

**Figure 2:**
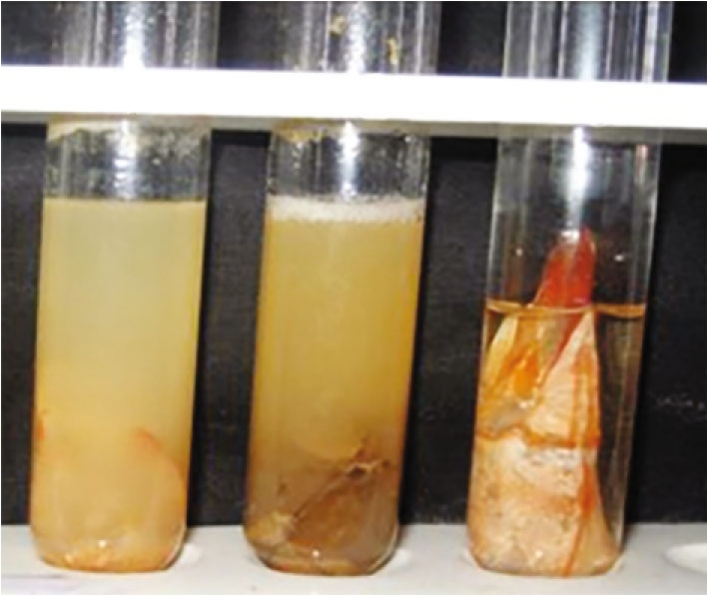
Shrimp shell degradation effects by partial purified chitinase enzyme produced by wild strains of *B*. *licheniformis* (SSCL-10). (1) Shrimp shells alone without *Bacillus licheniformis* SSCL10. (2) Shrimp shells with *Bacillus licheniformis* SSCL10 for 6 days. (3) Shrimp shells with *Bacillus licheniformis* SSCL10 for 12 days.

Figure 2 revealed the biodegradation of shrimp shells after 6 and 12 days in a time-dependent manner observed visually. Twelve days effectively degrades the exoskeleton of shrimp shell compared to 6 days.

### 3.2. UV Mutagenesis

**Figure 3:**
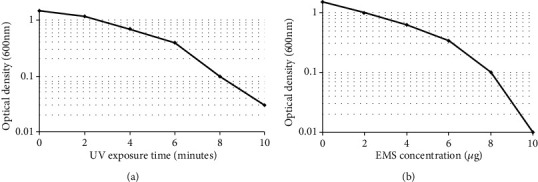
Survival curve of mutagenesis. (a) UV survival curve of *Bacillus licheniformis* SSCL-10. (b) EMS survival curve of *Bacillus licheniformis* SSCL-10 UV-11.

*Bacillus licheniformis* (SSCL-10) was mutated by UV and the survivors were plated on CCA plates to measure zone of clearance (CZ) to colony size (CS). Mutant hyperchitinase-producing strains were identified and isolated at different time intervals. The percentage survivors decreased in a dose-dependent manner at various time intervals (0, 2, 4, 6, 8, and 10 minutes) (Figure 3(a)). Thirteen mutants, which showed a high CZ/CS ratio, were examined for the production of chitinase with zones of clearance ranging from 15 to 19 mm in colloidal chitin agar medium. Mutant UV-11 was found to produce 13.3 U/mL (Table 1) of chitinase that was significantly higher (*p* < 0.05) than the wild type (3.4 U/mL) (Figure 1).

### 3.3. EMS Mutagenesis

*Bacillus licheniformis* (SSCL-10) mutant UV-11 was further mutated with EMS and the decrease in survivors was dose dependent (0-7 *μ*g) measured spectrophotometrically (Figure 3(b)). Sixteen mutants showing higher CZ/CS ratio were examined for the production of chitinase that gave zones of clearance ranging from 19 to 27 mm in a colloidal chitin agar medium. Mutant EMS 13 was found to produce the highest amount of chitinase of 20.2 U/mL (Table 1) that was significantly higher (*p* < 0.05) than the wild type (3.4 U/mL) and mutant UV-11 (13.3 U/mL) (Figure 1). The hyperchitinase yield of the mutant EMS-13 was measured and found to be stable by subculturing consecutively on CCA slants for over 6 months.

### 3.4. Specific Chitinolytic Activity

Figure 1 shows the analysis of chitinolytic activity among three significant mutant chitinase producers (*p* < 0.05) that have different chitinolytic capacities as compared to the wild strain. In the increasing order of specific activity, the wild *Bacillus licheniformis* (SSCL-10) produced the lowest followed by the mutant UV-11 and the highest was produced by the mutant EMS 13. The results of specific chitinase activity with the selected strains ranged from 3.4 U/mL to 20.4 U/mL of protein (Table 1) (Fig. S4). In addition, a strong association was found between the observed chitinase activity and the hydrolytic zone formation observed in each strain.

### 3.5. Phylogenetic Analysis

**Figure 4:**
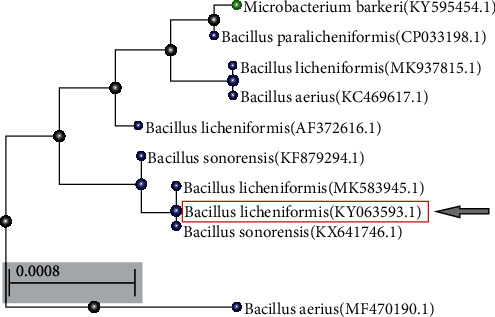
Phylogenetic tree of the selected strain *Bacillus licheniformis* SSCL-10 from other bacterial taxa.

Compared with the database sequences, the 16S rRNA from the SSCL-10 isolate showed 97% similarity with *Bacillus licheniformis* that was recorded in the NCBI database (Bank ID SUB2051105; Gene Bank Accession No. KY063593; identified organism *Bacillus licheniformis*) (Table S1-S2). The program Tree Dyn 198.3 was used for phylogenetic tree rendering (Table S3).  Figure 4 shows tree rendering results of *Bacillus licheniformis* (SSCL-10). The alignment and phylogenetic analysis of 16S rRNA sequences of different *Bacillus* species strongly suggested species status of the bacterial strain (SSCL-10) (Fig. S7) and confirmed the classification and identification of the isolated bacterial strains as *Bacillus licheniformis.*

### 3.6. Chitinase Purification

**Figure 5:**
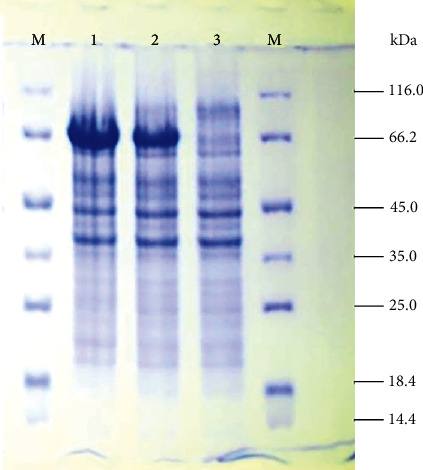
SDS-PAGE analysis for partial purification of chitinase from *Bacillus licheniformis* (SSCL-10). M: Marker, 1: cell extract fraction of wild-type strain, 2: cell extract fraction of UV mutant 11, 3: cell extract fraction of EMS-13.

The chitinase (Chi-66) was partially purified by 65% saturated ammonium sulfate (SAS) precipitation at 4°C. SDS-PAGE of the denatured partially purified chitinase identified the molecular weight near 66 kDa (Figure 5). Based on the equivalent amount of total protein loaded for all the strains, the precipitate of the wild type showed a thin, small band; the UV mutant strain revealed a medium size band; and the EMS mutant strain showed a thick band indicating the increasing degree of yield of chitinase (Fig. S5-S6). The EMS mutant chitinase-producing strain effectively produces the highest level of chitinase compared to UV mutant and wild type.

## 4. Discussion

Chitinase has recently been successfully isolated and characterized from *Bacillus* species [29–31]. However, it is necessary to produce high yield of chitinases to fulfill the essential needs for the sustenance of the ecosystem. The present research reports the isolation, molecular identification, and induced mutagenesis of high yielding wild strain of *Bacillus licheniformis* with chitinolytic activity from shrimp wastes. Our previous study reported chitinase-producing isolates, its characterization, and optimal growth factors such as temperature, pH, nutrient composition of the culture medium with colloidal chitin concentration, chitinase production, and its activity from different marine sources [7]. The chitinolytic activity was indicated by the presence of a zone of clearance given by the isolates. Earlier studies demonstrated that the presence of halo (zone of clearance) requires a long incubation period of 5 to 6 days [32]. Presence of Gram-negative isolates is common in the marine environment, especially in crustaceans and shrimp wastes that can cause diseases in marine organisms. Our earlier studies observed that chitinase produced by *Bacillus licheniformis* (SSCL-10) degrades exoskeleton of cockroach effectively [7]. Similarly, a present study also proved that the enzyme produced by *Bacillus licheniformis* (SSCL-10) effectively degrades shrimp shell wastes. Our results revealed that *Bacillus licheniformis* (SSCL-10) is a potential organism to be used as a biocontrol agent and improve ecofriendly environment.

Compared to Gram-positive bacteria, 90% of the diversity of Gram-negative bacteria in marine ecosystems has traits that enhance survival of these organisms in extreme temperature, tolerance and rapid adaptation to nutrient deficiencies, and high salt concentration [33, 34]. The chitinase activity of the mutated strains of UV-11 and EMS-13 showed values greater than earlier reports [27] with a maximum specific enzymatic activity of 14.2 U/mL in a strain of *Bacillus licheniformis* B307. On the other hand, our wild strain of *Bacillus licheniformis* SSCL-10 showed chitinolytic activity values of 3.4 U/mL, which is lower than the mutant values compared in this study and reported in our earlier studies [7]. Dai et al. [35] reported that *Bacillus* spp. Hu1 showed chitinase activity of 11.1 U/mg of protein in crude extract, whereas *B*. *licheniformis* LHH100 showed chitinase activity of 494.5 U/mg of protein reported by Laribi-Habchi et al. [15].

In addition to normal production of chitinase, we designed mutant strains for increased chitinase activity by inducing UV and EMS mutation. This method was applied in an earlier study with *Alcaligenes xylosoxydans*, which enhances the chitinase production 2-3-fold higher [26]. Our study is the first report of hyperchitinase activity in *Bacillus licheniformis* induced chemically (EMS) and by UV radiation. Furthermore, the UV mutant *Bacillus licheniformis* SSCL-10 UV-11 enhanced chitinase production 3-4-fold higher than the wild type. Further, chitinase production was enhanced by mutagenizing the UV mutant (UV-11) with EMS (EMS-13) with a specific activity of 20.4 U/mL. Thus, UV and chemical mutation induced higher yield of chitinase from the wild-type *Bacillus licheniformis* SSCL-10 by 5-6-fold. Others have reported twofold enhanced chitinase production in *Serratia marcescens* QMB 1466 [36] and in *Pseudomonas stutzeri* YPL-M26 with N-methyl-N′-nitro-N-nitrosoguanidine (NTG) mutagenesis [37]. The partially purified chitinase was identified as a 66 kDa (Figure 5) molecular weight protein by SDS-PAGE. Researchers have isolated and purified chitinase produced by *Bacillus* spp. with a range of 36, 42, 53, 59, 62, 66, 72, 76, and 89 kDa [38–40]. Chitinases have been isolated from other microorganisms with molecular weights falling in the same range. An endochitinase of 66 kDa was isolated and cloned from *Serratia proteamaculans* [41]. Another study found that four chitinases with molecular weights of 92, 82, 70, and 55 kDa secreted by *Aeromonas caviae* CB101 were encoded by a single gene *chi1* [42]. The proteins were different truncations of the same Chi1 protein possibly due to posttranslation proteolytic cleavage that forms the chitinase processing. However, the role of such processing is uncertain. Most of the chitinolytic bacteria studied have multiple chitinases that function synergistically for chitin degradation. Further elucidation of the chitinase structure from our study will highlight the similarities and differences with other chitinases and indicate possibly the presence of multiple truncations of the same protein. Our previous study reported that the isolated chitinase enzyme tested as important biocontrol agents against selected phytofungal pathogens viz. *Fusarium solani*, *Rhizconia solani*, *Fusarium oxysporum*, and *Aspergillus* spp. [18].

*Bacillus licheniformis*, a part of the subtilis group, is commonly found in soil and bird feathers. In birds, it is involved in feather degradability and impacts molting and color patterns. In humans, these commonly cause food poisoning and are a contaminant of dairy products, raw milk, vegetables, cooked meats, and processed baby foods. Since this bacterium produces and secretes hydrolytic enzymes, it has the ability to degrade many substrates. This degradability feature has captured the interest for potential applications in biotechnology [18, 43]. The fermented bird feathers are turned into nutritious livestock feed. It also produces an alkaline protease, which is in turn used in laundry detergent as it can remove proteinaceous dirt from clothes at lower temperatures. It is used as a biopesticide as it possesses antifungal activity [17, 44]. This has probiotic potential when used along with *B*. *subtilis* that promotes better immune function. *B*. *licheniformis* has a significant role to play in the bioconversion of chitin, one of the major wastes of the crustacean industry. Chitin is difficult to biodegrade that poses an environmental problem which can be alleviated by chitinases. Chitinases is responsible for the bioconversion of chitin to pharmacologically active products such as N-acetyl glucosamine and chito-oligosaccharides. These derivatives can eventually contribute to a sustainable environment and further downstream applications. The characterization and sequencing of *Bacillus licheniformis* could represent a biotechnological alternative to manipulate nonpathogenic microorganisms capable of decomposing chitin in marine ecosystem and for feasible industrial applications.

## 5. Conclusions

Chitinolytic potential was identified and isolated in *Bacillus licheniformis* (SSCL-10) from shrimp wastes. Our result revealed that *Bacillus licheniformis* (SSCL-10) was a potential organism to be used as a biocontrol agent and improve the ecofriendly environment. Mutagenesis was induced in the isolated wild strain to enhance the production of chitinase. Extracellular chitinase was partially purified, and the size of the purified enzyme was around 66 kDa. The chitinase yield was enhanced 6-7-fold in the mutant strains as compared to the wild strain. Sequencing of 16S rRNA of the isolated wild chitinase producer determined that it belongs to the species *Bacillus licheniformis*, and the sequence was recorded in the NCBI database (Bank ID SUB2051105; Gene Bank Accession No. KY063593; identified organism *Bacillus licheniformis*). The strain with the hyperchitinolytic capacity was mutant EMS-13, with a specific activity of 20.4 U/mL. The EMS-induced mutant strain from *Bacillus licheniformis* SSCL-10, EMS-13, was identified as a highest yielding chitinase producer that can find potential applications in biocontrol, pharmaceuticals, and biotechnological sectors (Fig. S1). Furthermore, future studies that include molecular analysis of the chitinase structure can help in drawing comparisons with other characterized chitinases. This will also help in studying further the possibility of a chitin-degradation system available in *Bacillus* and the presence of multiple chitinases or truncated forms of the same protein. Due to its high degradability feature, industrial applications for using this enzyme can be explored. Hyperchitinase mutants can be investigated for bioconversion of chitinous wastes at commercial level. Further, its biocontrol on pathogenic fungi can be employed for disease control among commercially important crops thereby minimizing the use of pesticides and its negative effects on crops and human health.

## Figures and Tables

**Table 1:** Ratio of zone of clearance (CZ)/size of colony (CS) and chitinase activity of *Bacillus licheniformis* SSCL-10 wild type, maximum CZ/CS of three mutants of UV and EMS strains were selected for chitinase production.

S. no.	Bacterial isolate	CZ/CS (after 48 h)	Chitinase activity (units/mL^−1^)
1	Wild-type strain	13-14	3.40 ± 0.31
2	UV mutant 4	16-17	10.8 ± 0.98
3	UV mutant 7	16-17	11.2 ± 1.14
4	UV mutant 11	18-19	13.3 ± 1.21
5	EMS mutant 6	22-23	18.7 ± 1.42
6	EMS mutant 9	22-23	18.1 ± 1.30
7	EMS mutant 13	24-25	20.2 ± 1.72

## Data Availability

All data used in this study available as Supplementary Material for this article can be found online.

## Additional Points

*Highlights.* (1) A chitinase producer was isolated and identified by 16S rRNA gene sequencing as *B*. *licheniformis*. (2) Mutagenesis by EMS and UV induced increased hyperchitinase production as compared to the wild chitinase production. (3) Chitinase was partially purified and determined as a 66 kDa protein. (4) This study offers an effective industrial-grade strategy for hyperchitinase production that can bioprocess nondegradable chitin wastes from environmental crustaceans and shrimp residues.

## Supplementary Materials

Supplementary Materials Figure 1: chitinolytic producers were isolated and identified from soil containing shrimp wastes. The isolate Bacillus licheniformis (SSCL-10) was tested for shrimp degradation properties and mutagenesis. Molecular typing of 16S rRNA sequence revealed the phylogenetic lineage of Bacillus licheniformis (SSCL-10). The wild strain was mutated with UV, and high-yielding chitinase producer (UV-11) was selected for EMS mutation and compared the chitinase production between wild and mutant strains. Extracellular chitinase was partially purified and the size of the purified enzyme was determined around 66 kDa. Our result indicated the potential of the organism as a biocontrol agent that can aid in improving an ecofriendly environment. Figure 2: primary screening and isolation of chitinase-degrading organism in colloidal chitin agar. Figure 3: morphology of wild strain B. licheniformis SSCL-10. Figure 4: chitinase activity of wild and mutant strains. Figure 5: HPLC of partially purified chitinase enzyme produced by Bacillus licheniformis (SSCL-10). Figure 6: partial purification of SDS-PAGE (original). Table 1: analysis report of B1 (B1_contig_1 report). Table 2: blast N report of B1. Table 3: parameters used for constructing the phylogenetic tree.
