# Supplementary material for: Identification and Characterization of a Newly Isolated Chitinase-Producing Strain Bacillus licheniformis SSCL-10 for Chitin Degradation
Source: Archaea. 2020 Nov 9;2020:8844811. doi: 10.1155/2020/8844811 (PMC7669355; doi:10.1155/2020/8844811)
Supplement: Supplementary Materials — Figure 1: chitinolytic producers were isolated and identified from soil containing shrimp wastes. The isolate Bacillus licheniformis (SSCL-10) was tested for shrimp degradation properties and mutagenesis. Molecular typing of 16S rRNA sequence revealed the phylogenetic lineage of Bacillus licheniformis (SSCL-10). The wild strain was mutated with UV, and high-yielding chitinase producer (UV-11) was selected for EMS mutation and compared the chitinase production between wild and mutant strains. Extracellular chitinase was partially purified and the size of the purified enzyme was determined around 66 kDa. Our result indicated the potential of the organism as a biocontrol agent that can aid in improving an ecofriendly environment. Figure 2: primary screening and isolation of chitinase-degrading organism in colloidal chitin agar. Figure 3: morphology of wild strain B. licheniformis SSCL-10. Figure 4: chitinase activity of wild and mutant strains. Figure 5: HPLC of partially purified chitinase enzyme produced by Bacillus licheniformis (SSCL-10). Figure 6: partial purification of SDS-PAGE (original). Table 1: analysis report of B1 (B1_contig_1 report). Table 2: blast N report of B1. Table 3: parameters used for constructing the phylogenetic tree. [file 8844811.f1.pdf]

Figure 1: Graphical Abstract

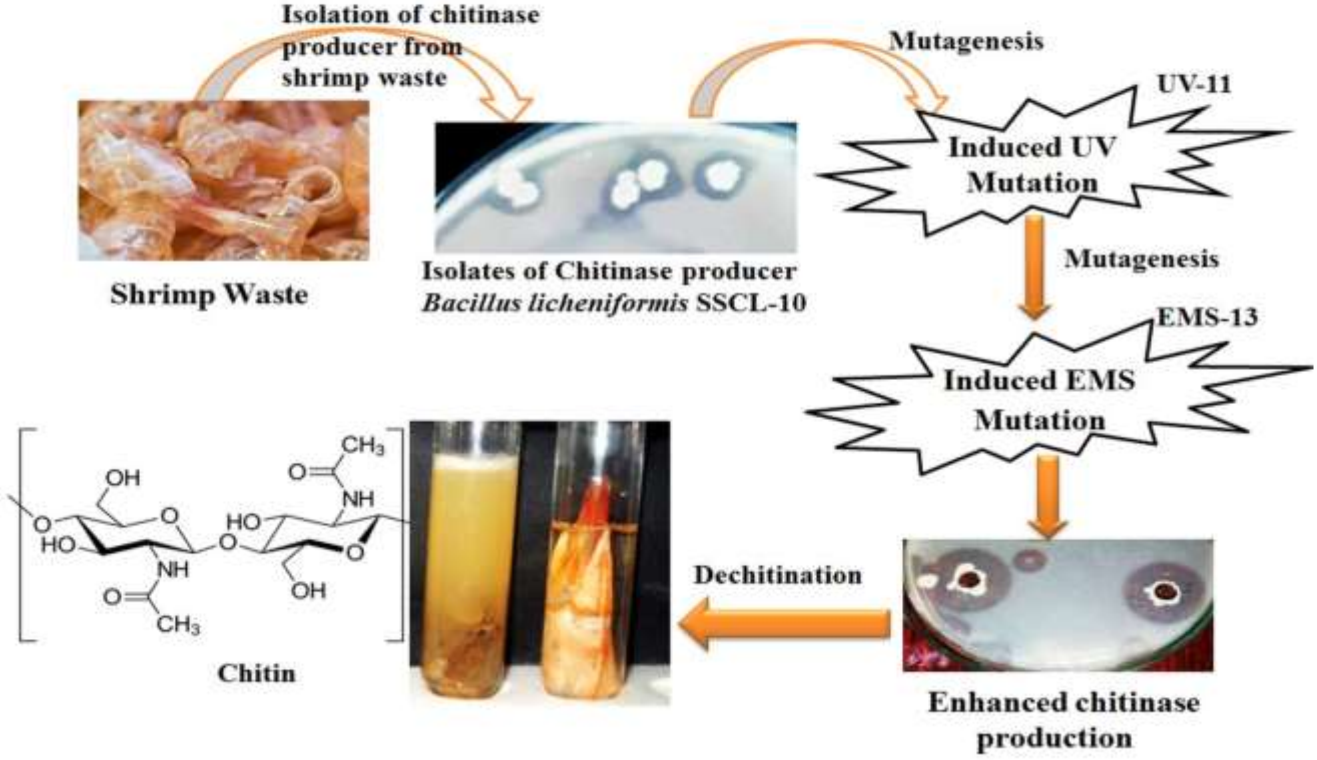

Figure 1 Chitinolytic producers were isolated and identified from soil containing shrimp wastes. The isolate *Bacillus licheniformis* (SSCL-10) was tested for shrimp degradation properties and mutagenesis. Molecular typing of 16S rRNA sequence revealed the phylogenetic lineage of *Bacillus licheniformis* (SSCL-10). The wild strain was mutated with UV and high-yielding chitinase producer (UV-11) was selected for EMS mutation and compared the chitinase production between wild and mutant strains. Extracellular chitinase was partially purified and the size of the purified enzyme was determined around 66kDa. Our result indicated the potential of the organism as a biocontrol agent that can aid in improving an eco-friendly environment.

Figure: 2 Primary screening and isolation of chitinase degrading organism in colloidal chitin Agar

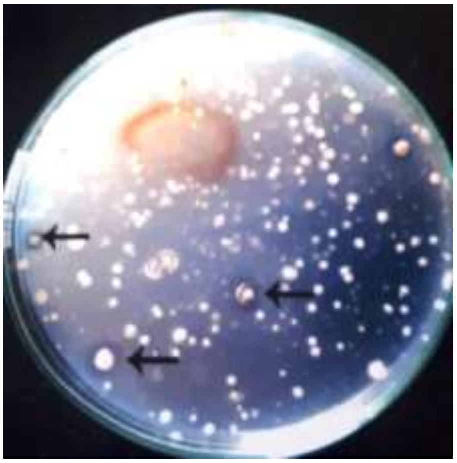

The soil samples were collected from dumping sites of shrimp shell waste at Thoothukudi, Tamil Nadu, India. 10g of soil sample was collected carefully in an aseptic condition and the samples were placed in a sterile container and kept on ice till it reaches the laboratory. The primary screening was performed by spot inoculation of isolates on colloidal chitin agar plates to identify the chitinase producer and incubated at 37°C for two days. Primary screening isolated 16 different chitinase producers identified by measuring the zone of clearance in the colloidal chitin agar.

Figure: 3 Morphology of wild strain *B. licheniformis* SSCL-10

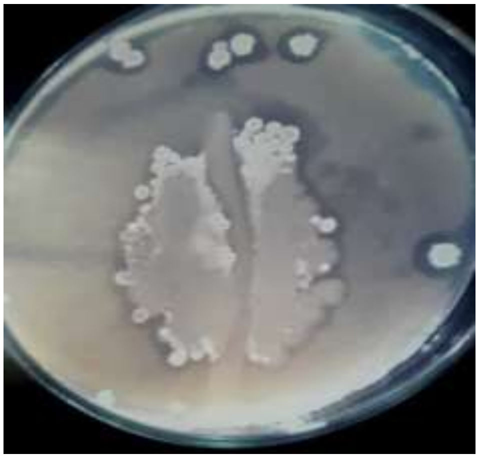

Among 16 different chitinase producers identified from primary screening the high yielding strain was selected for further analysis. The high yield strain was inoculated into colloidal chitin agar plate for morphology analysis. Figure 3 shows the colony morphology of *Bacillus licheniformis* (SSCL-10) as white, circular, smooth texture and convex shape with full borders.

Microscopic examination confirmed rod shaped, motile, vegetative and endospore forming cells that belong to *Bacillus* group.

Figure: 4 Chitinase activity of wild and mutant strains

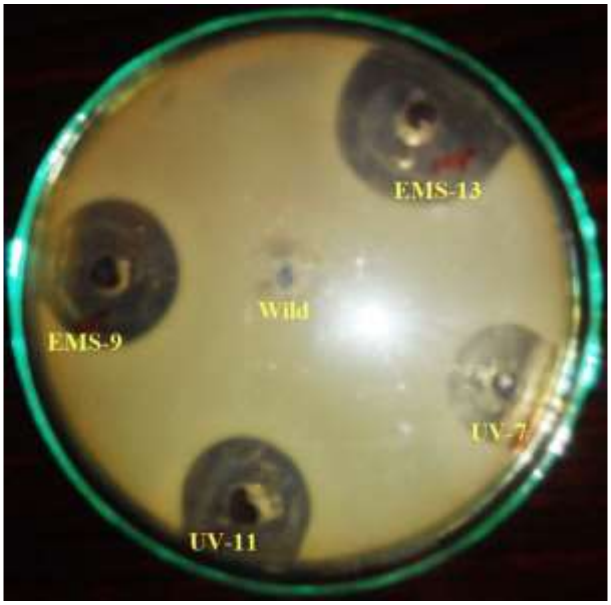

Secondary screening for chitinase enzyme was determined with the culture filtrates of the *Bacillus licheniformis* (SSCL-10), UV-11 and EMS-13 using well diffusion method. The wild and mutant strains were grown in 1% colloidal chitin nutrient broth. One ml of wild and mutant strains inoculum with OD 0.5 were inoculated to 100 ml of 1% colloidal chitin minimal medium and incubated for 48 h at 40°C in a rotary shaker at 100 rpm. The wild and mutant culture filtrates were collected by centrifugation at 10,000 rpm for 15 minutes. The culture filtrate of each isolates (100 µL) were placed in 6 mm well of 1% colloidal chitin agar plates and incubated at 37°C for 24 hrs. Figure 4 revealed chitinase enzyme activity by the development of clear zone around the well on colloidal chitin agar plates. The chitinase activity was observed by *B. licheniformis* (SSLC 10) wild, UV-7, UV-11, EMS-9 and EMS-13. Among the wild and mutant chitinase producers, EMS-13 zone was found to produce the highest amount of chitinase (units/ml) as mentioned in the text.

Figure: 5 HPLC of partially purified chitinase enzyme produced by *Bacillus licheniformis* (SSCL-10).

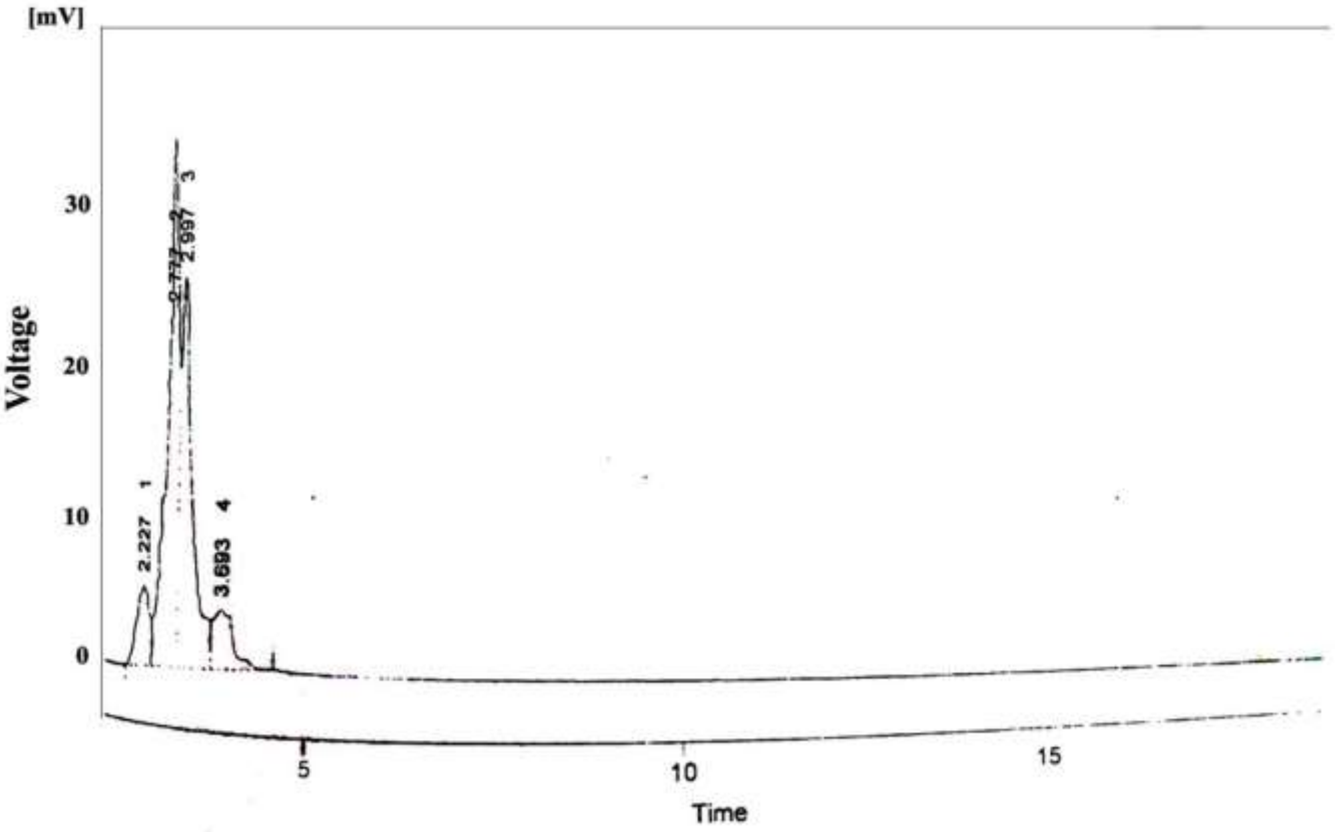

	Retention time	Area [mV.s]	Height [mV]	Area [%]	Height [%]	WOS [min]
1.	2.227	103.896	5.348	9.7	7.7	0.35
2.	2.777	484.933	34.931	45.4	50.1	0.22
3.	2.997	390.875	25.611	36.6	36.8	0.21
4.	3.693	88.844	3.773	8.3	5.4	0.37
	Total	10.58.547	69.662	100.0	100.0	

HPLC of chitinase enzyme extracted from *Bacillus licheniformis* (SSCL-10) gave four peaks; among them two were higher. The degradation of chitin polysaccharides results in oligosaccharides and monosaccharides. The peak may represent to oligosaccharides.

Figure: 6 Partial purification of SDS-PAGE (Original)

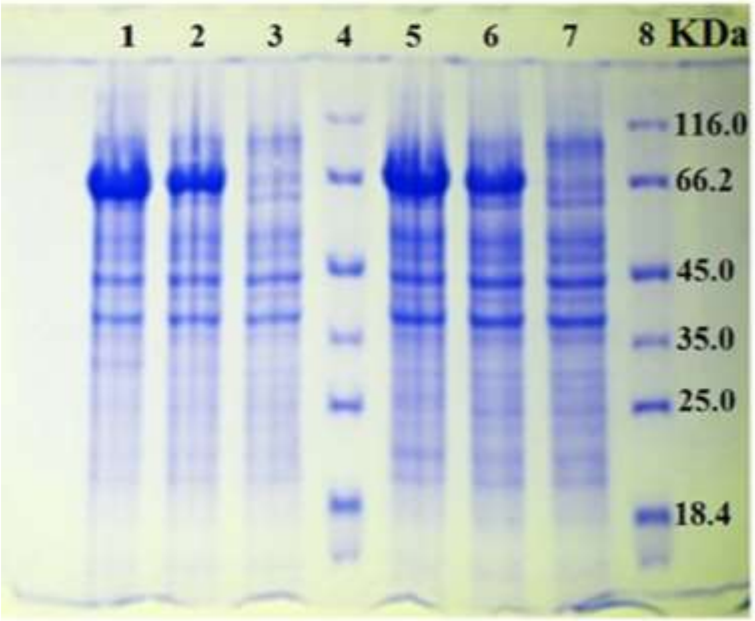

Chitinase produced by wild and mutant strains were partially purified and molecular weight was estimated by SDS-PAGE as 66 KDa. Lane 1: Cell extract fraction of EMS-13, Lane 2: Cell extract fraction of UV-mutant-11, Lane 3: Cell extract fraction of Wild Type strain, Lane 4: Marker, Lane 5: Cell extract fraction of Wild Type strain, Lane 6: Cell extract fraction of UV-mutant 11, Lane 7: Cell extract fraction of EMS-13, Lane 8: Marker. See supplementary Figure 1 for full image.

Table 1-3 and Figure 7 revealed the molecular typing (16S rRNA)and phylogenetic tree construction of chitinase producer

Table 1: Analysis Report of B1 (B1_contig_1 Report)

**Name**	**Read Length (Normal)**	**Read Length (Q16)**	**Read Length (Q20)**	**GC Content**
**B1_contig_1**	**1524**	**1438**	**1436**	**54.7244094488189**
B1_F	689	684	682	54.862119013062404
B1_R	895	872	867	54.63687150837989

Figure 7. Sequence of *Bacillus licheniformis* SSLC-10

TTTTTAGAGGTTATTACACAGCTCAGGACGAACGCTGGCGGCGTGCCTAATACATGC
AAGTCGAGCGGACAGATGGGAGCTTGCTCCCTGATGTTAGCGGCGGACGGGTGAGT
AACACGTGGGTAACCTGCCTGTAAGACTGGGATAACTCCGGGAAACCGGGGCTAAT
ACCGGATGCTTGATTGAACCGCATGGTTCAATTATAAAAGGTGGCTTTTAGCTACCA
CTTACAGATGGACCCGCGGCGCATTAGCTAGTTGGTGAGGTAACGGCTCACCAAGG
CGACGATGCGTAGCCGACCTGAGAGGGTGATCGGCCACACTGGGACTGAGACACGG
CCCAGACTCCTACGGGAGGCAGCAGTAGGGAATCTTCCGCAATGGACGAAAGTCTG
ACGGAGCAACGCCGCGTGAGTGATGAAGGTTTTCGGATCGTAAAACTCTGTTGTTAG
GGAAGAACAAGTACCGTTCGAATAGGGCGGTACCTTGACGGTACCTAACCAGAAAG
CCACGGCTAACTACGTGCCAGCAGCCGCGGTAATACGTAGGTGGCAAGCGTTGTCC
GGAATTATTGGGCGTAAAGCGCGCGCAGGCGGTTTCTTAAGTCTGATGTGAAAGCCC
CCGGCTCAACCGGGGAGGGTCATTGGAAACTGGGGAACTTGAGTGCAGAAGAGGAG
AGTGGAATTCCACGTGTAGCGGTGAAATGCGTAGAGATGTGGAGGAACACCAGTGG
CGAAGGCGACTCTCTGGTCTGTAACTGACGCTGAGGCGCGAAAGCGTGGGGAGCGA
ACAGGATTAGATACCCTGGTAGTCCACGCCGTAAACGATGAGTGCTAAGTGTTAGA
GGGTTTCCGCCCTTTAGTGCTGCAGCAAACGCATTAAGCACTCCGCCTGGGGAGTAC
GGTCGCAAGACTGAAACTCAAAGGAATTGACGGGGGCCCGCACAAGCGGTGGAGC
ATGTGGTTTAATTCGAAGCAACGCGAAGAACCTTACCAGGTCTTGACATCCTCTGAC
AACCCTAGAGATAGGGCTTCCCCTTCGGGGGCAGAGTGACAGGTGGTGCATGGTTG
TCGTCAGCTCGTGTCGTGAGATGTTGGGTTAAGTCCCGCAACGAGCGCAACCCTTGA
TCTTAGTTGCCAGCATTCAGTTGGGCACTCTAAGGTGACTGCCGGTGACAAACCGGA
GGAAGGTGGGGATGACGTCAAATCATCATGCCCCTTATGACCTGGGCTACACACGT
GCTACAATGGGCAGAACAAAGGGCAGCGAAGCCGCGAGGCTAAGCCAATCCCACA
AATCTGTTCTCAGTTCGGATCGCAGTCTGCAACTCGACTGCGTGAAGCTGGAATCGC
TAGTAATCGCGGATCAGCATGCCGCGGTGAATACGTTCCCGGGCCTTGTACACACCG
CCCGTCACACCACGAGAGTTTGTAACACCCGAAGTCGGTGAGGTAACCTTTTGGAGC
CAGCCGCCGAAGGTGGGACAGATGATTGGGGTGAAGTCGTAACAGGGAAACCCGTA
AAA

Table 2: Blast N Report of B1

- Query name : B1_contig_1

- Query length : 1524

**Query**		**Subject**				**Score**			**Identities**			
**Start**	**End**	**AC**	**Length**	**Start**	**End**	**Bit**	**Raw**	**EV**	**Match**	**Total**	**Pct. (%)**	**Strand**
21a	1517	AF372616.1	1509	13	1509	2754	1491	0.0	1495	1497	99	Plus/Plus
21b	1509	CP005965.1	4376305	34106	35594	2750	1489	0.0	1489	1489	100	Plus/Plus
21c	1522	KC469617.1	1516	13	1513	2750	1489	0.0	1498	1502	99	Plus/Plus
21[d]	1517	IIM006901.1	1511	15	1511	2748	1488	0.0	1494	1497	99	Plus/Plus
21[e]	1507	KF917167.1	1504	3	1489	2747	1487	0.0	1487	1487	100	Plus/Plus
21[f]	1509	DQ993676.1	1538	15	1503	2747	1487	0.0	1488	1489	99	Plus/Plus
21[g]	1509	KFO55012.1	1534	14	1502	2745	1486	0.0	1488	1489	99	Plus/Plus
21[h]	1509	EU718490.1	1549	20	1508	2745	1486	0.0	1488	1489	99	Plus/Plus
21[i]	1509	AY728013.1	1549	14	1502	2745	1486	0.0	1488	1489	99	Plus/Minus
21[j]	1522	KC429647.1	1516	16	1516	2743	1485	0.0	1496	1502	99	Plus/Plus

Description :

a-*Bacillus licheniformis* strain Mol 16S ribosomal RNA gene, partial sequence, **b**-*Bacillus licheniformis* 9945A *Complete genome*, **c**-*Bacillus cereus* strain RGS230 16S ribosomal RNA gene, partial sequence, **d**-*Bacillus licheniformis* strain Ph-WC 09001 16S ribosomal RNA gene, partial sequence, **e**-*Bacillus sp.* BAB-3437 16S ribosomal RNA gene, partial sequence, **f**-*Bacillus licheniformis* strain BCRC 15413 16S ribosomal RNA gene, partial sequence, **g**-*Bacillus subtilis* strain IARI-JR-68 16S ribosomal RNA gene, partial sequence, **h**-*Bacillus licheniformis* strain MSS-14- 16S ribosomal RNA gene, partial sequence, **i**-*Bacillus licheniformis* strain JM4 16S ribosomal RNA gene, partial sequence, **j**-*Bacillus licheniformis* strain XFB-AK 16S ribosomal RNA gene, partial sequence. Based on the description, the identified organism was *Bacillus licheniformis*.

Table 3 Parameters used for constructing phylogenetic tree

**Parameters**
Minimum number of sequences for a conserved position: 4<br>Minimum number of sequences for a flanking position: 5<br>Maximum number of contiguous non-conserved positions: 8<br>Minimum length of a block: 10<br>Allowed gap positions: none<br>**Flank positions of the 1 selected block(s)**<br>Flanks: [22 1517]<br>New number of positions in input.fasta-gb: **1496** (97% of the original 1527 positions)

The isolated wild chitinase producer identified as *Bacillus licheniformis* was based on morphological and biochemical characteristics features. Further species identification of *Bacillus licheniformis* strain SSCL10 was confirmed using 16S rRNA gene sequencing. The 16s rRNA sequence was mapped against NCBI blast similarity search tool (Accession No. KY063593) (Table 1). The analysis report of B1 (B1_contig_1 Report) of *Bacillus licheniformis* has been presented in Table 2. The BLASTN search of the *Bacillus licheniformis* obtained 4376305 nucleotide sequences which showed representative homology with *Bacillus* species with maximum homology of 100% with *Bacillus licheniformis* strain C19945A (Accession No. CP005965.1). Molecular typing of 16S rRNA sequence of isolated organism was identified as *Bacillus licheniformis* shown in Figure 7. The parameter used for constructing phylogenetic tree is shown in Table 3. The program Tree Dyn 198.3 was used for tree rendering. The phylogeny analysis of our sequence with the closely related sequence of blast results was performed followed by multiple sequence alignment. The alignment and phylogenetic analysis of 16S rRNA sequences of different *Bacillus* species strongly suggested species status of the bacterial strain SSCL10 as *Bacillus licheniformis.*
